# Wearable Humidity Sensor Using Cs_3_Cu_2_I_5_ Metal Halides with Hydroxyl Selective Phase Transition for Breath Monitoring

**DOI:** 10.3390/bios15050311

**Published:** 2025-05-13

**Authors:** Si Hyeok Yang, Lim Kyung Oh, Dong Ho Lee, Donghoon Gwak, Nara Song, Bowon Oh, Na Young Lee, Hongki Kim, Han Seul Kim, Jin Woo Choi

**Affiliations:** 1 Department of Data Information and Physics, Kongju National University, Gongju-si 32588, Republic of Korea; sihuck015@gmail.com (S.H.Y.); dh35329@gmail.com (D.G.); narragood@gmail.com (N.S.); ojs7935@naver.com (B.O.); ny07049@gmail.com (N.Y.L.); 2 Department of Advanced Materials Engineering, Chungbuk National University, Cheongju-si 28644, Republic of Korea; dhdlarud1209@naver.com (L.K.O.); dh.lee@chungbuk.ac.kr (D.H.L.); 3 Department of Chemistry, Kongju National University, Gongju-si 32588, Republic of Korea; 4 Earth Environment Research Center, Kongju National University, Gongju-si 32588, Republic of Korea; 5 Department of Urban, Energy, Environmental Engineering, Chungbuk National University, Cheongju-si 28644, Republic of Korea; 6 Institute of Application and Fusion for Light, Kongju National University, Gongju-si 32588, Republic of Korea

**Keywords:** low-dimensional metal halides, gas sensor, wearable device, health care

## Abstract

The low-dimensional metal halide Cs_3_Cu_2_I_5_ exhibits unique electrical and chemical properties. Notably, it undergoes a phase transition to CsCu_2_I_3_ upon exposure to hydroxyl (-OH) gas, resulting in significant changes in its electrical characteristics. In this study, we developed a highly selective semiconductor-based gas sensor utilizing Cs_3_Cu_2_I_5_. The material was synthesized on an Al_2_O_3_ substrate with carbon electrodes using a solution-based process, enabling gas sensing based on its electrical properties. The sensor was further integrated into an Arduino-based real-time monitoring system for wearable applications. The final system was mounted onto a face mask, enabling the real-time detection of human respiration. This research presents a next-generation sensor platform for real-time respiratory monitoring, demonstrating the potential of Cs_3_Cu_2_I_5_ in advanced wearable bio-gas sensing applications.

## 1. Introduction

The Fourth Industrial Revolution is being driven by artificial intelligence (AI), and high-quality data are essential for machine learning and its performance improvement [1,2,3,4]. In particular, various environmental data can be obtained through different sensors [5,6,7,8]. In this context, the development of sensor technology has emerged as a critical area of research, driven by the need for reliable high-quality data collection [9,10]. Among the various sensors, gas sensors provide essential environmental information for applications such as air quality monitoring, industrial safety, and healthcare applications, which are also crucial in an aging society that accompanies the Fourth Industrial Revolution [11,12,13,14,15]. These applications are becoming important in an aging society to accompany the Fourth Industrial Revolution, with a particular focus on breath analyses using gas sensors and electronic noses [16,17]. Conventional gas detection techniques, including gas chromatography, mass spectrometry, and atomic absorption spectrometry, offer high accuracy but suffer from limitations such as high costs, bulky instrumentation, and the need for trained operators, making them impractical for real-time, on-site applications [18,19,20,21].

To overcome these challenges, cost-effective and integrable semiconductor gas sensors have been developed [22,23,24,25]. These semiconductor gas sensors operate based on changes in electrical properties induced by gas adsorption and desorption [26,27,28,29]. However, if there is a lack of gas desorption on the surface, high operating temperatures are required to vaporize the adsorbed gases. Therefore, active materials are exposed to air at high temperatures, leading to oxidation [30]. To prevent device degradation, the material choices are limited to oxide semiconductors. The emergence of two-dimensional (2D) semiconductor materials, such as graphene, has opened new possibilities by enabling gas detection at room temperature with enhanced sensitivity [31,32]. Nevertheless, the commercialization of 2D semiconductor-based sensors remains challenging due to the high fabrication costs and selectivity among the various gas molecules. Hence, an innovative approach would be to develop novel semiconductor materials that are operable at room temperature while exhibiting high stability and selectivity.

Perovskite-type semiconductor materials have emerged as promising candidates for gas sensors due to their defect-tolerant semiconducting properties and low-cost solution processability [33,34]. Through the successful introduction of active layers, sensors for various gases such as NH_3_, NO_2_, H_2_S, and CO have been developed [35,36]. However, despite the demonstrated electrical and optical responses of perovskite materials to gases, their stability and selectivity remains a challenge. Due to the instability of the halide perovskite structure caused by ionic bonding in the presence of moisture and heat, stable and highly responsive sensor performance with a groundbreaking sensing mechanism has been actively pursued.

Recently, we successfully developed a highly selective Cs_3_Cu_2_I_5_/CsCu_2_I_3_ semiconductor gas sensor, based on a novel reversible phase transition mechanism [37]. Cs_3_Cu_2_I_5_ exhibits a reversible phase transition from Cs_3_Cu_2_I_5_ to CsCu_2_I_3_ when exposed to -OH functional groups, leading to reversible electrical conductivity and optical emission characteristics during gas adsorption and desorption. A gas sensor based on this phase transition mechanism demonstrates over one-year stability, maintaining 90% of its initial responsibility. However, the practical utilization of these materials is challenging without an additional breakthrough, primarily due to the limitation on the real-time response caused by the phase transition time. Additionally, although selectivity was achieved based on chemical reactivity, it was shown that the Cs_3_Cu_2_I_5_ reacts to several materials with OH groups. However, it is noteworthy that a 40-fold difference in reactivity between H₂O (HOH) and methanol (MeOH) was observed. This suggests that by adjusting the measurement range, the response characteristics to MeOH could be treated as noise, allowing for the development of a high-selectivity gas sensor that responds exclusively to H_2_O.

In this study, we propose a wearable semiconductor-based gas sensor system utilizing a Cs_3_Cu_2_I_5_ phase transition active material, with high sensitivity to H_2_O. The Cs_3_Cu_2_I_5_ material was synthesized on an Al_2_O_3_ substrate with carbon electrodes using a solution-based process, facilitating its integration into practical sensor devices. Furthermore, to overcome the limitation in response time due to the requirement for a chemical reaction, a transistor-based signal amplification system is employed using an Arduino sensor system. This approach is expected to improve the response time from the previously reported 60 s to 2~4 s, making it more suitable for respiratory monitoring. To demonstrate its feasibility, the sensor system was mounted onto a face mask, enabling the real-time detection of human respiration. This research introduces an innovative gas sensor platform that not only enhances the gas detection sensitivity and selectivity but also expands the potential of Cs_3_Cu_2_I_5_ for advanced wearable bio-gas sensing applications.

## 2. Materials and Methods

Materials: The cesium iodide (CsI, 99.999%), copper(I) iodide (CuI, 99.995%), and *N,N*-dimethylformamide (99.8%) were purchased from Sigma-Aldrich (Louis, MO, USA). The methanol, ethanol, and 2-propanol were purchased from Samchun Chemicals (Daegu, Republic of Korea). All chemicals were used without further purification.

Fabrication of the Cs_3_Cu_2_I_5_: CsI and CuI were dissolved in DMF at a molar ratio of 3:2 to prepare the 2.0 M precursor solutions. The precursor was stirred at 60 °C for 24 h inside a glove box. After the stirring process, the solutions were filtered through PTFE filters with a pore size of 0.45 µm to remove impurities.

Gas Sensor Device Fabrication: The gas sensor device was fabricated on aluminum oxide substrates. Carbon electrodes were deposited on the substrate using the silk printing method. The substrates were cleaned using sonication in deionized (DI) water, acetone, and IPA for 15 min. The Cs_3_Cu_2_I_5_ film was prepared by spin-coating the precursor solution at 1000 rpm for 60 s, followed by thermal treatment at 100–150 °C for 1 h.

Gas Sensor System: The response and recovery times were set by measuring the steady-state response of the detective gases under 1.5 V bias at room temperature with a homemade system, as shown in Figure S3. The electrical measurements were conducted using the Keithley2601-B source meter.

Breath Analysis Using Wearable Sensor: For real-time humidity monitoring and breath analyses, the sensor was integrated into an Arduino-based system and embedded in a mask to monitor humidity variations during different physical activities.

Characterization Methods: The phase of Cs_3_Cu_2_I_5_ was confirmed via XRD (MiniFlex II, Rigaku, Tokyo, Japan) with scan angles ranging between 10° and 60°. The PL spectra were obtained using an PL spectrometer (CCS200/M, Thorlabs, Newton, NJ, USA), with excitation provided by a 285 nm laser. The absolute PLQY was measured using a Hamamatsu Quantus-QY spectrometer. The crystalline morphology of the Cs_3_Cu_2_I_5_ films deposited on the substrate was examined using SEM (S-4700 FE-SEM, HITACHI, Tokyo, Japan). The electrical properties of the sensor were characterized using a Keithley 2601-B source meter, measuring the current response.

Computational Methods: First-principles calculations based on DFT were performed using the Vienna Ab Initio Simulation Package (VASP) to obtain the relaxed atomic structures of Cs_3_Cu_2_I_5_. The projector augmented wave (PAW) method was employed with norm-conserving pseudopotentials, considering the Cs (5s, 5p), Cu (3d, 4s), and I (5s, 5p) states explicitly [38]. A plane-wave basis set with a cutoff energy of 520 eV was used to ensure accurate wavefunction representation. Exchange correlation interactions were described using the Perdew–Burke–Ernzerhof revised for solids (PBEsol) functional within the generalized gradient approximation (GGA) [39,40]. Brillouin zone integration was performed using a Γ-centered 2 × 2 × 1 k-point grid for unit–cell calculations. Ionic relaxation was conducted until the total energy difference between successive ionic steps was less than 2 × 10⁻⁶ eV, ensuring structural convergence.

## 3. Results and Discussion

**Figure 1:**
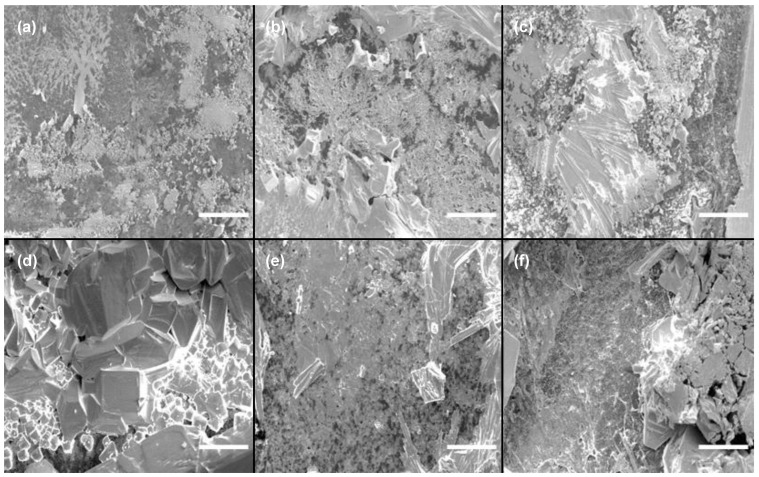
SEM images of Cs_3_Cu_2_I_5_ thin films deposited on Al_2_O_3_, thermally treated at different temperatures: (**a**) 100 °C; (**b**) 110 °C; (**c**) 120 °C; (**d**) 130 °C; (**e**) 140 °C; (**f**) 150 °C. The inset scale bar represents 50 μm.

The Cs_3_Cu_2_I_5_ active thin film was deposited on an Al_2_O_3_ substrate using a solution process. Based on our previous studies, it was found that Cs_3_Cu_2_I_5_ exhibits a sensitive coverage dependency on substrate materials and is even significantly affected by the orientation of the glass substrate surface [37]. Such poor coverage not only increases defects, which degrade semiconductor properties, but also leads to a lack of a percolation path between electrodes, potentially preventing the sensor from functioning properly. Therefore, scanning electron microscopy (SEM) was used first to analyze the morphology of the deposited coatings on surfaces. In particular, to investigate the coverage characteristic of Cs_3_Cu_2_I_5_ on the Al_2_O_3_ thin film under different thermal treatment temperatures, the growth temperature was adjusted from 100 °C to 150 °C. Examples of SEM images of the thin films at different temperatures are presented in Figure 1. 

**Figure 2:**
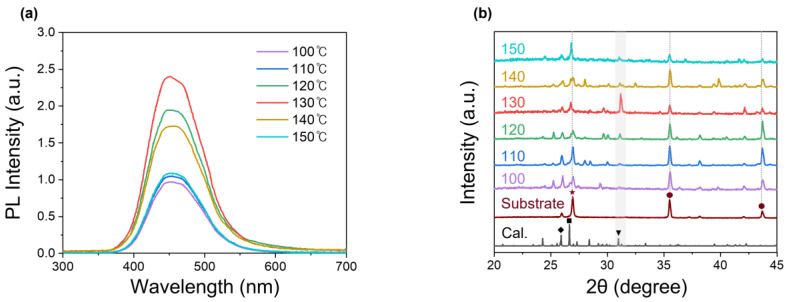
Characteristics of Cs_3_Cu_2_I_5_ thin films treated at different temperatures: (**a**) PL spectra; (**b**) XRD patterns.

The film surfaces changed significantly, even with a 10 °C variation in thermal treatment temperature. A notable observation was the presence of a rippled surface grain structure in the low-temperature region. In samples prepared at 100 °C to 120 °C, the slow evaporation of *N,N*-dimethylformamide (DMF) at lower temperatures provided sufficient time for solute movement due to the Marangoni effect during thin film formation. As a result, a wavy pattern was observed, accompanied by poor coverage and substrate exposure. In contrast, in the high-temperature region, such as at temperatures above 140 °C, the rapid evaporation rate resulted in the formation of small-sized polycrystalline crystals on the order of a few micrometers, along with some intermediate-sized crystals (~30 μm). At 130 °C, the conditions provided sufficient time and thermal energy for crystal growth, leading to the formation of well-developed crystals with sizes of around 50 μm. Overall, considering the coverage and excellent crystal properties, 130 °C was identified as the optimal annealing temperature, as it produced the largest and most well-formed crystals. For a better characterization of Cs_3_Cu_2_I_5_ thin films, photoluminescence (PL) and X-ray diffraction (XRD) analyses were performed (Figure 2).

Figure 2a shows the PL characteristics of the thin films under 285 nm laser excitation at different thermal treatment temperatures. Previous studies have reported that Cs_3_Cu_2_I_5_ single crystals exhibit a photoluminescence quantum yield (PLQY) of 91%, while thin films show a 62% yield, with emissions occurring at 450 nm.

This difference in PLQY values between single crystals and thin films has been attributed to the relatively lower crystallinity of thin films [41]. Similarly, in this study, all synthesized Cs_3_Cu_2_I_5_ thin films exhibited blue emissions with a peak wavelength centered at 450 nm. However, variations in peak intensity were observed. These changes were likely due to exciton annihilation caused by the trap density. As confirmed by the SEM images, the Cs_3_Cu_2_I_5_ sample treated at 130 °C, which had the largest crystal size, also exhibited the best PL characteristics, with a PLQY of 55%. For a better understanding of the crystallinity of Cs_3_Cu_2_I_5_ thin films, a set of density functional theory (DFT) simulations and XRD analyses were performed (Figure 2b). In the XRD results, the diffraction peaks for crystal planes of Cs_3_Cu_2_I_5_ structure were well matched with those of the DFT simulation. The peaks at 25.9°, 26.7°, and 31.0° corresponded to the (−1 2 3), (2 −2 −2), and (0 4 0) planes of Cs_3_Cu_2_I_5_ and were marked with a diamond, square, and triangle, respectively. Additionally, the Al_2_O_3_ substrate peaks were also observed at 25.4°, 35.0°, and 43.8°. The main peaks 35.0° and 43.8° are indicated by circles [42]. Additionally, the carbon bottom electrode peak detected at 26.5° is marked with a star [43]. Among the three Cs_3_Cu_2_I_5_ main peaks, the 31.0° peak was selected to evaluate the crystallinity, as it did not overlap with peaks from the underlying substrate and electrode. At 130 °C, the peak intensity reached its maximum, indicating the formation of relatively large crystallites and suggesting optimal crystal growth and improved crystallinity compared to lower temperatures such as 110 °C. Furthermore, the high thermal treatment temperatures (e.g., 150 °C) resulted in lower XRD intensity compared to the sample treated at 130 °C, due to the formation of small polycrystalline domains. Therefore, this result confirms that the best crystallinity is achieved at 130 °C, which also correlates well with the PL characteristics observed in Figure 2a. Additionally, as observed in the SEM images (Figure 1), at lower temperatures, the substrate exposure resulted in stronger substrate peaks and a broad underlying amorphous peak ranging from 15° to 35° in the XRD patterns.

**Figure 3:**
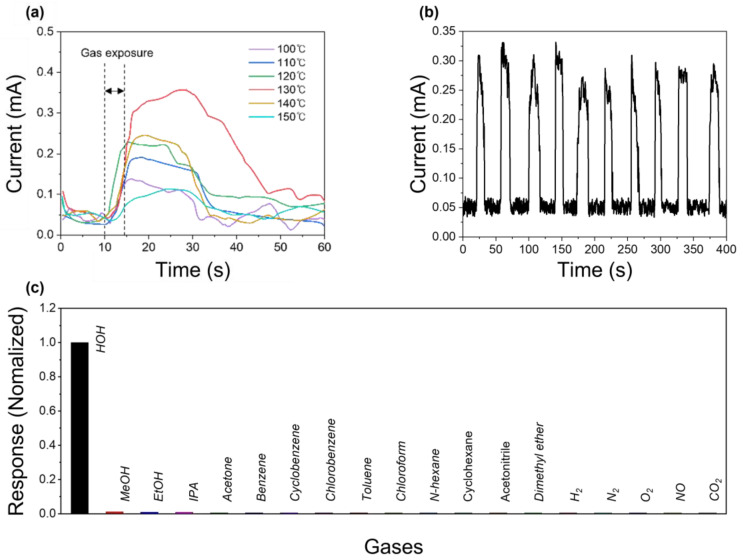
(**a**) Electrical response of the Cs_3_Cu_2_I_5_ gas sensor fabricated at different thermal treatment temperatures. (**b**) Electrical response of the Cs_3_Cu_2_I_5_ gas sensor under repeated H_2_O on–off exposures. (**c**) Selectivity of the sensor under exposure to various gases at room temperature.

Figure 3a presents the gas detection characteristic of the Cs_3_Cu_2_I_5_-based gas sensor with different thermal treatment conditions. The sensor was placed in a gas chamber, linked to various gas generators. The gas flow rate was controlled at 200 sccm, and the concentration of vaporized molecules carried by the flow gas was measured and calibrated using commercial gas sensors. The electrical response was measured using Keithley 2601-B equipment. 

The response to H_2_O gas was measured, and as expected from the SEM, PL, and XRD results, the sample annealed at 130 °C exhibited the best responsiveness on the inlet gas. The repeatability characteristics of the Cs_3_Cu_2_I_5_ gas sensor fabricated at 130 °C are shown in Figure 3b. On average, the rising time (T_r_) was 4 s and the falling time (T_f_) was 10 s. Figure 3c presents the gas detection selectivity of the Cs_3_Cu_2_I_5_-based gas sensor. 

The electrical response remained unchanged under exposure to various gases, including MeOH, ethanol (EtOH), 2-propanol (IPA), H_2_, N_2_, O_2_, CO_2_, and NO. Moreover, volatile organic compounds (VOCs) such as acetone, benzene, cyclobenzene, chlorobenzene, toluene, chloroform, n-hexane, and cyclohexane were undetectable through electrical responses. Even polar solvents, including acetonitrile and dimethyl ether, did not induce crystal structure transformation, confirming the high selectivity of the developed gas sensor toward H_2_O. Interestingly, this gas sensor did not respond to methanol or ethanol. This phenomenon aligns with previous findings, where the reaction sensitivity to H_2_O was reported to be 40 times higher than methanol, which ranks second in reactivity [37]. In previous sensors fabricated with gold electrodes, featuring a 100 μm interdigitated length and 10 interdigitated electrode pairs, methanol detection was possible due to high-sensitivity equipment and an advanced electrode structure [37]. However, in this study, the carbon electrodes fabricated using a silk printing method had a significantly wider interdigitated gap of 500 μm and contained only 4 electrode pairs, reducing their sensitivity. As a result, while the methanol induced some degree of Cs_3_Cu_2_I_5_ crystal structure transformation, its effect was significantly weaker than that of H_2_O, lowering the signal to the noise level (Figure S1). Thus, while the introduction of a carbon electrode clearly sacrifices the overall sensor signal magnitude, such an approach ensures enhanced selectivity toward H_2_O detection.

**Figure 4:**
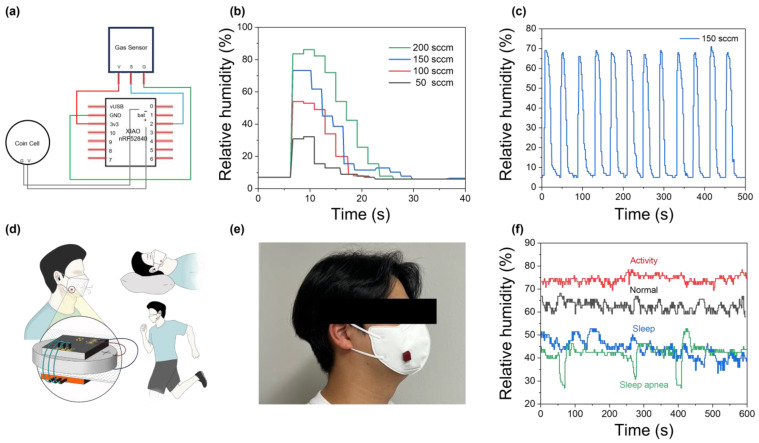
(**a**) Arduino circuit diagram of the fabricated system. (**b**) Humidity response of the Cs_3_Cu_2_I_5_ gas sensor system at different flow rates. (**c**) Repeated operation characteristics of the Cs_3_Cu_2_I_5_ gas sensor. (**d**) Schematic illustration of the wearable gas sensor mask. (**e**) Actual wearing demonstration. (**f**) Breathing pattern analyses in various environments such as normal breathing, activity, sleep, and sleep apnea.

To harness the selectivity of the carbon electrode while addressing its low signal magnitude, a transistor-based signal amplification system was introduced, making the sensor compatible with commercial sensor using Arduino (Figure 4a). Figure 4b,c show the gas sensor’s operation and repeatability characteristics in a real-time measurement system using Arduino. For the numerical validation of the developed system, the measured humidity values were calibrated using a commercial sensor. As shown in Figure S2, the T_r_ and T_f_ of the widely used commercialized DHT11 sensor in conventional Arduino systems are 6 s and 25 s, respectively. In comparison, in Figure 4b and Figure S2b, the sensor developed in this study exhibited T_r_ and T_f_ values of 2 and 6 s, demonstrating a superior response speed compared to commercial sensors. From the perspective of the repeatability characteristics, the sensor exhibited a slightly faster average response time compared to the Keithley-based measurements shown in Figure 3. This difference is likely due to the increased responsibility provided by the transistor components in the system. To compare the performance of our sensor with previously reported humidity sensors, a summary is provided in Table S1. Finally, an integrated respiratory monitoring system was implemented by embedding Arduino nrf52480 into the wearable mask, as shown in Figure 4d,e. Using this system, as shown in Figure 4f, human breathing patterns were analyzed under various conditions. During intense physical activity, the overall relative humidity level increased to approximately 80% due to rapid breathing, which was noticeably higher compared to the typical level of 65%. Additionally, during sleep, the measured humidity level of the breathing was relatively lower, at around 50%. Interestingly, a significant decrease in humidity was observed during sleep apnea, followed by recovery. Generally, sleep apnea is characterized by an individual experiencing approximately five instances of apnea per hour during sleep, each lasting for at least 10 s, followed by a deep gasp for breath. This repetitive pattern was successfully measurable with the developed wearable system. This capability demonstrates its potential for wearable healthcare applications, as changes in breath humidity are closely associated with respiratory health conditions such as sleep apnea [44]. Future studies could further expand the field of wearable healthcare by incorporating additional gas sensors or modifying the sensors into fiber-based forms for enhanced integration.

## 4. Conclusions

In this study, a Cs_3_Cu_2_I_5_ gas sensor with high selectivity for H_2_O detection was developed and optimized. SEM, PL, and XRD analyses confirmed that an annealing temperature of 130 °C resulted in the best crystallinity and gas response. The sensor exhibited excellent selectivity, showing no response to other -OH group gases such as methanol or ethanol, as well as VOCs and organic solvents. The gas sensor was successfully integrated into an Arduino-based wearable gas sensor mask for real-time respiratory monitoring. The sensor effectively distinguished between different breathing patterns, demonstrating its potential for wearable healthcare applications. Future studies could enhance its functionality by integrating additional gas sensors or adapting fiber-based designs for improved wearability and flexibility. 

## Data Availability

The original contributions presented in this study are included in the article. Further inquiries can be directed to the corresponding author.

## Supplementary Materials

The following supporting information can be downloaded at https://www.mdpi.com/article/10.3390/bios15050311/s1: Figure S1. Electrical response of the Cs_3_Cu_2_I_5_ gas sensor to methanol (MeOH). Figure S2. The T_r_ and T_f_ of the (a) DHT11 sensor and (b) Cs3Cu2I5 gas sensor under 150 sccm of H_2_O vapor. Figure S3. Schematic illustration of gas sensor system configuration. Table S1. The performance parameters of the reported humidity sensors. References [45,46,47,48,49,50] are cited in the Supplementary Materials.
