# Supplementary material for: Wearable Humidity Sensor Using Cs3Cu2I5 Metal Halides with Hydroxyl Selective Phase Transition for Breath Monitoring
Source: Biosensors (Basel). 2025 May 13;15(5):311. doi: 10.3390/bios15050311 (PMC12110226; doi:10.3390/bios15050311)
Supplement: Supplementary file 1 [file biosensors-15-00311-s001.zip › biosensors-3583287-supplementary.pdf]

biosensors

Supplementary Materials

Wearable Humidity Sensor Using $Cs_3Cu_2I_5$ Metal Halides with Hydroxyl Selective Phase Transition for Breath Monitoring

Si Hyeok Yang [1,†], Lim Kyung Oh [2,†], Dong Ho Lee [2,†], Donghoon Gwak [1,†], Nara Song [1], Bowon Oh [1], Na Young Lee [1], Hongki Kim [3,4,*], Han Seul Kim [2,5,*] and Jin Woo Choi [1,6,*]

[1] Department of Data Information and Physics, Kongju National University, Gongju-si 32588, Republic of Korea; sihuck015@gmail.com (S.H.Y.); dh35329@gmail.com (D.G.); narragood@gmail.com (N.S.); ojs7935@naver.com (B.O.); ny07049@gmail.com (N.Y.L.)
[2] Department of Advanced Materials Engineering, Chungbuk National University, Cheongju-si 28644, Republic of Korea; dhdlarud1209@naver.com (L.K.O.); dh.lee@chungbuk.ac.kr (D.H.L.)
[3] Department of Chemistry, Kongju National University, Gongju-si 32588, Republic of Korea
[4] Earth Environment Research Center, Kongju National University, Gongju-si 32588, Republic of Korea
[5] Department of Urban, Energy, Environmental Engineering, Chungbuk National University, Cheongju-si 28644, Republic of Korea
[6] Institute of Application and Fusion for Light, Kongju National University, Gongju-si 32588, Republic of Korea
* Correspondence: hongkikim@kongju.ac.kr (H.K.); hanseul.kim@chungbuk.ac.kr (H.S.K.); jinwoo.choi@kongju.ac.kr (J.W.C.)
† These authors contributed equally to this work.

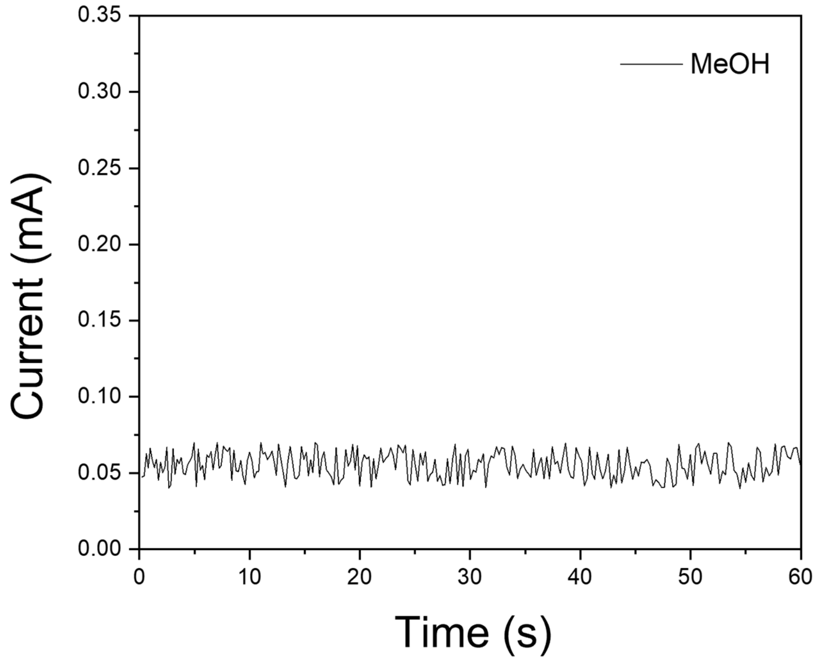

Figure S1. Electrical response of the $Cs_3Cu_2I_5$ gas sensor to methanol (MeOH).

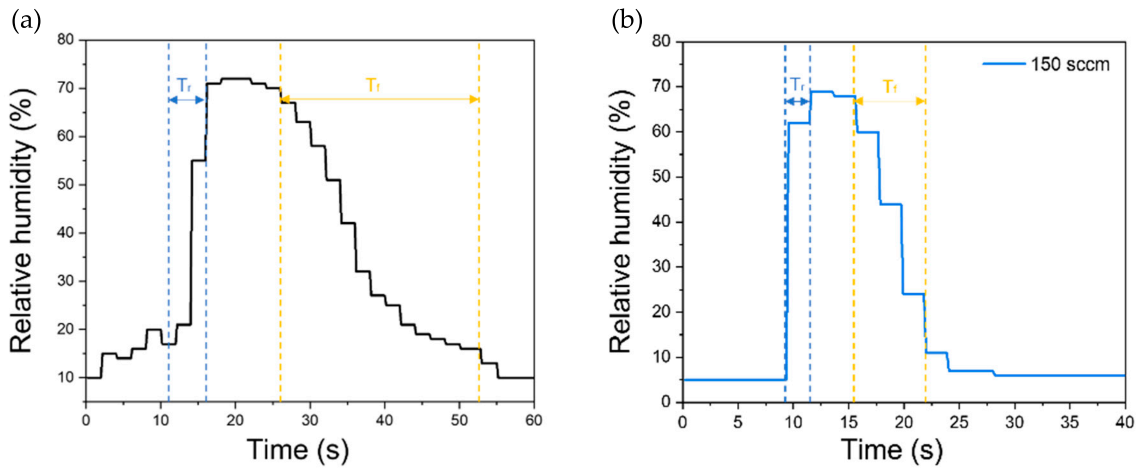

Figure S2. The rising time (T_r) and falling time (T_f) of the (a) DHT11 sensor and (b) $Cs_3Cu_2I_5$ gas sensor under 150 sccm of H_2O vapor.

Table S1. The performance parameters of reported humidity sensors

Materials	RH Range	Response/Recovery time	Ref
CNF/CB/TX-100	30–90%	10 s / 6 s	[1]
o-MWCNTs	33–95%	300 s/ 420 s	[2]
GNPS/MWCNT	20-90%	0.35 s / 2.5 s	[3]
Silk fibroin	59-95%	73.1 s / 11.3 s	[4]
CNT/PI	20-90%	480 s / 600 s	[5]
PEG/Gold Nanoparticle	1.8-95%	1.2 s / 3 s	[6]
$Cs_3Cu_2I_5$	20-90%	2 s / 6 s	This work

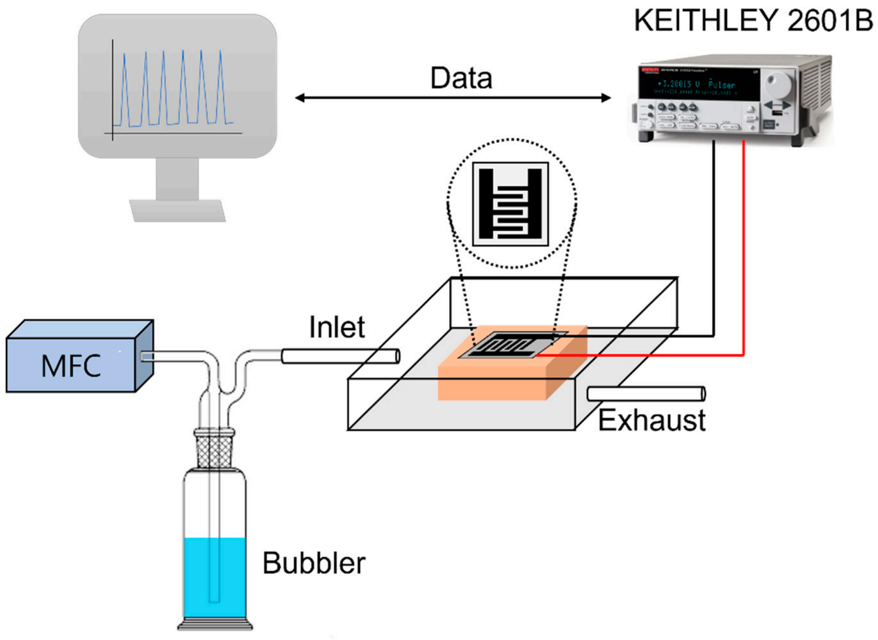

Figure S3. Schematic illustration of gas sensor system configuration.

References

1. Tachibana, S.; Wang, Y.-F.; Sekine, T.; Takeda, Y.; Hong, J.; Yoshida, A.; Abe, M.; Miura, R.; Watanabe, Y.; Kumaki, D.; Tokito, S. A Printed Flexible Humidity Sensor with High Sensitivity and Fast Response Using a Cellulose Nanofiber/Carbon Black Composite. *ACS Appl. Mater. Inter-faces* 2022, 14, 5721–5728. https://doi.org/10.1021/acsami.1c20918.
2. Zhao, H.; Zhang, T.; Qi, R.; Dai, J.; Liu, S.; Fei, T. Drawn on Paper: A Reproducible Humidity Sensitive Device by Handwriting. *ACS Appl. Mater. Interfaces* 2017, 9, 28002–28009. https://doi.org/10.1021/acsami.7b05181.
3. Chen, X.; Ma, K.; Ou, J.; Mo, D.; Lian, H.; Li, X.; Cui, Z.; Luo, Y. Fast-Response Non-Contact Flexible Humidity Sensor Based on Direct-Writing Printing for Respiration Monitoring. *Biosensors* 2023, 13, 792. https://doi.org/10.3390/bios13080792.
4. Zheng, Y.; Wang, L.; Zhao, L.; Wang, D.; Xu, H.; Wang, K.; Han, W. A Flexible Humidity Sensor Based on Natural Biocompatible Silk Fibroin Films. *Adv. Mater*. Technol. 2021, 6, 2001053. https://doi.org/10.1002/admt.202001053.
5. Tang, Q.-Y.; Chan, Y. C.; Zhang, K. Fast Response Resistive Humidity Sensitivity of Polyi-mide/Multiwall Carbon Nanotube Composite Films. *Sens. Actuators B Chem*. 2011, 152, 99–106. https://doi.org/10.1016/j.snb.2010.09.016.
6. Su, C.-H.; Chiu, H.-L.; Chen, Y.-C.; Yesilmen, M.; Schulz, F.; Ketelsen, B.; Vossmeyer, T.; Liao, Y.-C. Highly Responsive PEG/Gold Nanoparticle Thin-Film Humidity Sensor via Inkjet Printing Technology. *Langmuir* 2019, 35, 3256–3264. https://doi.org/10.1021/acs.langmuir.8b03433.
